# High-efficiency high-voltage class F amplifier for high-frequency wireless ultrasound systems

**DOI:** 10.1371/journal.pone.0249034

**Published:** 2021-03-29

**Authors:** Kyeongjin Kim, Hojong Choi

**Affiliations:** Department of Medical IT Convergence Engineering, Kumoh National Institute of Technology, Gumi, Republic of Korea; Karunya Institute of Technology and Sciences, INDIA

## Abstract

This paper presents a novel amplifier that satisfies both low distortion and high efficiency for high-frequency wireless ultrasound systems with limited battery life and size. While increasing the amplifier efficiency helps to address the problems for wireless ultrasound systems, it can cause signal distortion owing to harmonic components. Therefore, a new type of class F amplifier is designed to achieve high efficiency and low distortion. In the amplifier, the resonant circuit at each stage controls the harmonic components to reduce distortion and improve efficiency. Transformers with a large shunt resistor are also helpful to reduce the remaining noise in the input signal. The proposed class F amplifier is tested using simulations, and the voltage and current waveforms are analyzed to achieve correct operation with adequate efficiency and distortion. The measured performance of the class F amplifier has a gain of 23.2 dB and a power added efficiency (PAE) of 88.9% at 25 MHz. The measured DC current is 121 mA with a variance of less than 1% when the PA is operating. We measured the received echo signal through the pulse-echo response using a 25-MHz transducer owing to the compatibility of the designed class F amplifier with high- frequency transducers. The measured total harmonic distortion (THD) of the echo signal was obtained as 4.5% with a slightly low ring-down. The results show that the low THD and high PAE of the new high-efficiency and high-voltage amplifier may increase battery life and reduce the cooling fan size, thus providing a suitable environment for high-frequency wireless ultrasound systems.

## Introduction

**Fig 1:**
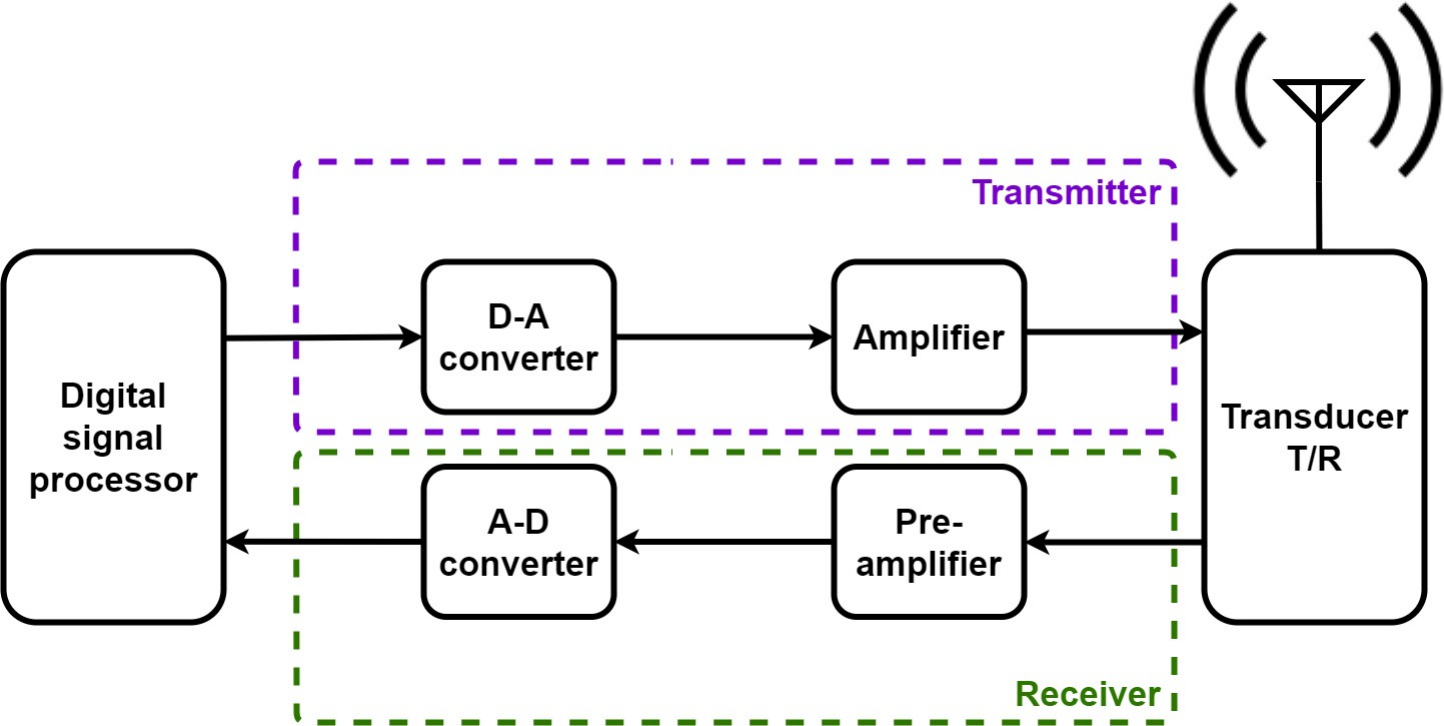
Wireless ultrasonic system block diagram [7,14,15].

Ultrasounds are widely used because of their many useful features such as low cost and real-time characteristics in everyday life. Their use is expected to further increase in the near future in areas such as diagnosis, screening, prevention, treatment, and protection devices [1–4]. Among them, many ultrasound imaging studies are performed which accounts for 25% of all imaging studies worldwide [5]. One of the reasons is that the non-destructive, non-invasive characteristics of ultrasound are very attractive characteristics in medical images as diagnostic tools. In addition, diagnostic devices that generate ultrasound images are in high demand because they are useful in the diagnosis of internal tissues and blood flow characteristics of small animals, the human body, or structures because the real-time inspection method is simple and the examination can be completed in a short period of time. In addition, there is no risk of radiation exposure [6,7]. However, most commercial ultrasound imaging devices have cables containing at least several dozen coaxial lines to power the transducer or share data between the transducer and the ultrasonic (ultrasound) system [8,9]. This type of cable is thick and long, obstructing scanning work, and its heavy weight can strain the arms and shoulders of users [10]. It is important to overcome such a fundamental disadvantage of ultrasound imaging diagnostic equipment. Wireless ultrasound imaging devices have a compact design with high portability and no cable restrictions, which is very useful for use in emergencies in various environments, and their use reduces the burden on the arms and shoulders of the users [11]. Therefore, they are expected to be attractive to doctors who usually work 24-h shifts in emergency rooms or outdoors, and the supply of home-care ultrasound cosmetic systems will see an increased demand by non-professional users who do not require medical diagnostic systems for physicians [12]. However, for the development of wireless ultrasound imaging systems that are comparable to conventional bench-top ultrasound imaging systems, it is necessary to consider the quality of the wireless ultrasound image that enables accurate diagnosis, and to maintain an adequate power efficiency for long-term use; this has implications for the portable battery that is used [13]. In Fig 1, a block diagram of a fundamental wireless ultrasonic (ultrasound) system is used to illustrate the system components.

The ultrasonic system consists of several components as shown in Fig 1 [7,14,15]. The amplifier in the transmitter station plays an important role in amplifying the signal needed for transmission to the transducer [1]. Because this kind of amplifier consumes a lot of power, the efficiency of amplifiers needs to be improved, thus reducing the power demand of wireless ultrasound systems. However, there are several considerations when designing such high-efficient high-voltage amplifiers. First, a high-voltage transistor used for a highly efficient amplifier in a switch mode can cause various problems [16]. For example, the switching behavior and nonlinear properties of high-voltage transistors distort the output signals because they produce the higher harmonics of the fundamental frequency [17]. The distorted output signals with harmonics can provide unwanted information and negatively affect image quality [18]. Therefore, it is necessary to increase the linearity by increasing the bias point or to remove the harmonic components of the amplifier by using a filter circuit [19]. However, raising and lowering the bias point are in trade-off relationship between efficiency and linearity, hence we need to adjust them appropriately. In addition, the amplifiers used in wireless ultrasound systems generate unnecessary heat for several reasons such as power loss, high current and etc. [20]. The heat of the transistor can change the performance of the amplifier during operations [21]. Therefore, to ensure the stable operation of such wireless ultrasound systems in any environment, the DC current used in the amplifiers should be reduced and temperature variances should be minimized using external cooling systems [11,22].

Image quality can be improved by increasing the frequency bandwidth of the transmission signal. In ultrasound systems, the higher the bandwidth, the shorter the spatial pulse length and better the resolution [2,17]. The improved detailed resolution clearly distinguishes the various targets in the image, and the shortened spatial pulse length improves the axial resolution [23]. However, ultrasound at high frequencies experiences an attenuation factor inside the medium, reducing the required depth of penetration for the target medium [24]. As a result, the target of the high-frequency ultrasound image appears clearer, but the attenuation factor of the medium is more dominant so the distance to be penetrated by the signal is smaller [25]. Recently, a resolution enhancement compression (REC) method was developed to improve axial resolution, bandwidth, and the echo signal-to-noise ratio (eSNR) using the coded excitation and pulse compression technique [26]. Strengthening the eSNR using REC improves the image quality and penetration depth [26,27]. As these technologies can increase the penetration depth, a high-frequency ultrasound system that can obtain clear images will be needed. While high-frequency ultrasound systems are sufficiently available, most commercial products currently have frequencies below 15 MHz.

The class E and class F amplifiers, which are used primarily for mobile or wireless applications, were proposed to implement high-efficiency amplifiers [28,29]. The class E amplifier is simpler than class F amplifier in tuning resonators. In class F, the resonator for picking third harmonic is a very narrow band and requires delicate tuning [30,31]. However, by controlling the third harmonics through the resonator of class F amplifier, the impedance of the third harmonics can be greatly increased [32]. As a result, low THD can be achieved by using class F topology. In addition, the power density of the class E amplifier is lower than the class F amplifier. Therefore, the output power of the class E amplifier is lower than that the class F amplifier [33]. The ultrasonic transducers in the wireless ultrasonic systems still require high output power. Hence, high power density design is necessary to obtain more triggering power at less space through class F topology. The class F amplifier achieves high efficiency through the switching operation of the transistor [21]. It can achieve a low distortion rate by clearly controlling certain harmonic components using a parallel resonator. Furthermore, a larger number of harmonics can be controlled by adding a parallel resonator, and higher efficiency can be achieved by changing the output waveform of the voltage and current. However, the design complexity of the class F amplifier circuits is proportional to the performance efficiency [34,35]. As a result, the class F amplifier can be balanced by varying the complexity, efficiency, and distortion rates depending on the designer. In addition, class F amplifiers can fine-tune and adjust certain harmonic components compared with class E amplifiers, so class F amplifier models that are suitable for wireless ultrasound systems can be implemented.

### Class F amplifier theory and simulation

To design a high-efficiency and high-voltage class F amplifier, we simulated and analyzed the class F amplifier using the software Advanced Designed System (ADS).

#### Efficiency of two-stage high-efficiency and high-voltage class F amplifier

**Fig 2:**
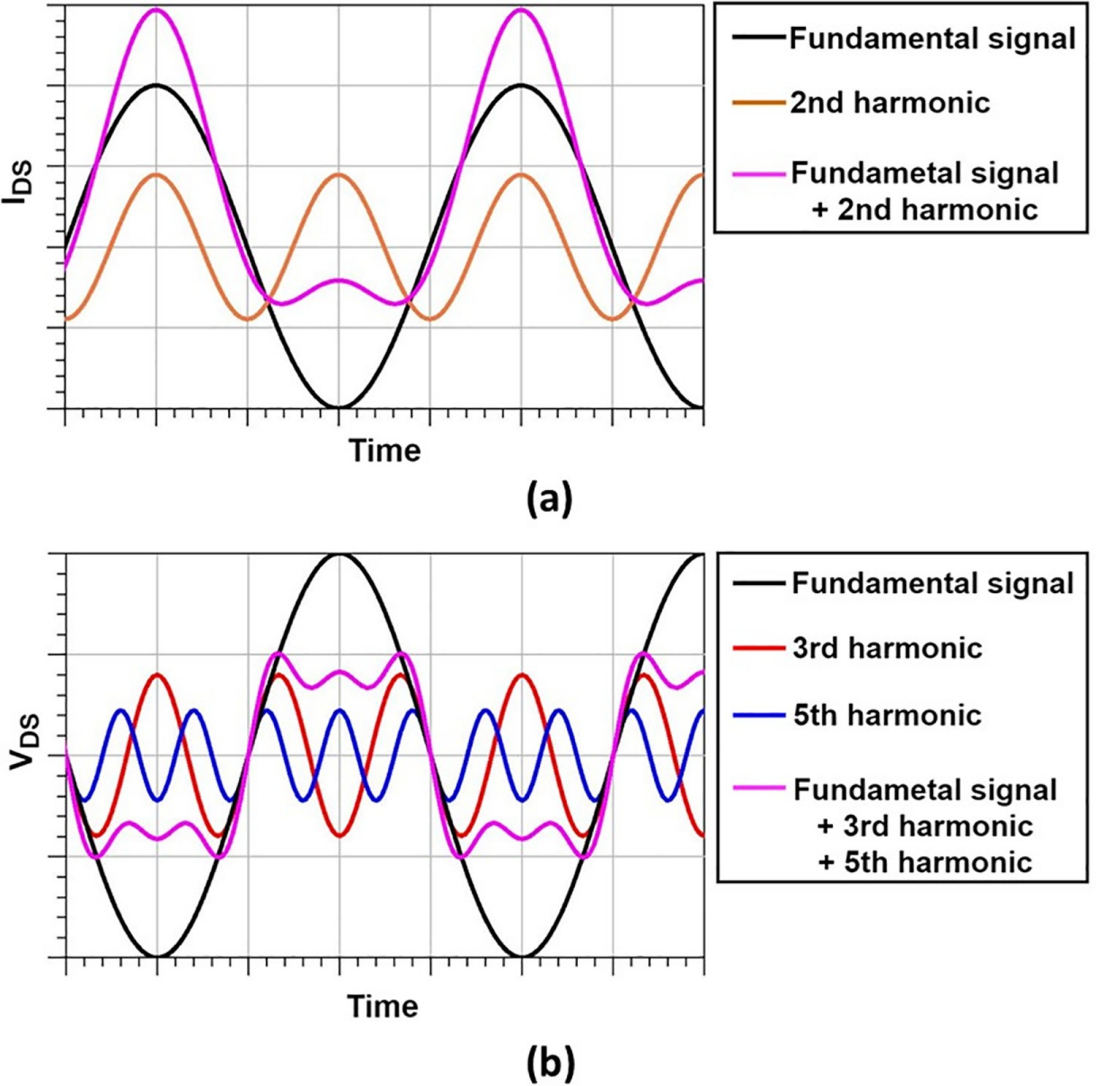
The principles by which waveforms are transformed by harmonics. The V_DS_ is formed by the original signal and the odd harmonic, and the I_DS_ is formed by the original signal and the even harmonic.

The class F amplifier controls harmonics through the output network to minimize the phase overlap of the current and voltage, possibly reducing power consumption between the drain and source of the high-voltage transistor [36]. The current is transformed as shown in Fig 2(A) by even harmonics, and the voltage is transformed as shown in Fig 2(B) by odd harmonics. From the results, the efficiency of the system can be maximized by minimizing the power consumption of the amplifier [34,35]. The drain voltage gradually flattens as odd harmonics are combined with the fundamental signal. When all odd harmonics are controlled, the drain source voltage and current becomes a completely flat waveform similar to the characteristic graph of an ideal class F amplifier (see Fig 2).

**Fig 3:**
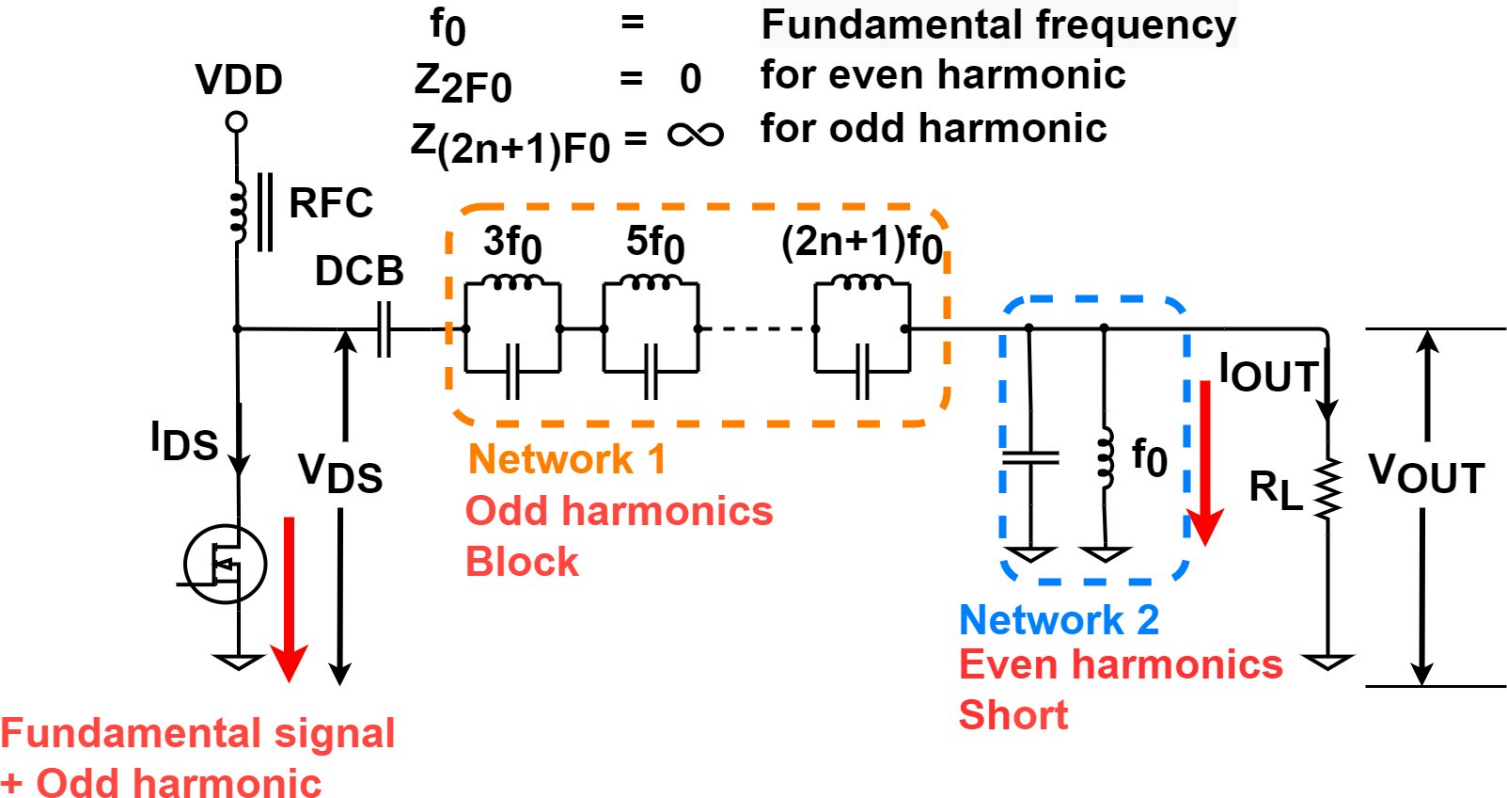
Typical network roles for a class F amplifier.

The design shown in Fig 3 was used to design a class F amplifier. Network 1 blocks odd harmonics and operates as an open-circuit, while Network 2 operates short-circuits for even harmonics. To design an ideal class F amplifier, all odd harmonics must be blocked [37]. Whenever an odd harmonic is controlled, network 1 is required, and infinite serial network 1 is required to control all odd harmonics. Therefore, it is virtually impossible to control all odd harmonics because of the need for infinite space and devices. In general, considering the complexity and efficiency of space and equipment use, it is common to control up to the third harmonic [35]. This is because the additional harmonic control helps very little efficiency improvement due to the various losses that occur in practice. The amplifier in this paper deals with 25 MHz, so our case is lower than microwave (frequency equal to 300 MHz to 300 GHz). Therefore, few losses from high frequency. However, the two-stage amplifier occurs greater losses in practice. Compared to ideal case, as a result, additional harmonic control will not help a significant efficiency improvement. Therefore, in this paper, the Class F amplifier was controlled up to the third harmonic.

**Fig 4:**
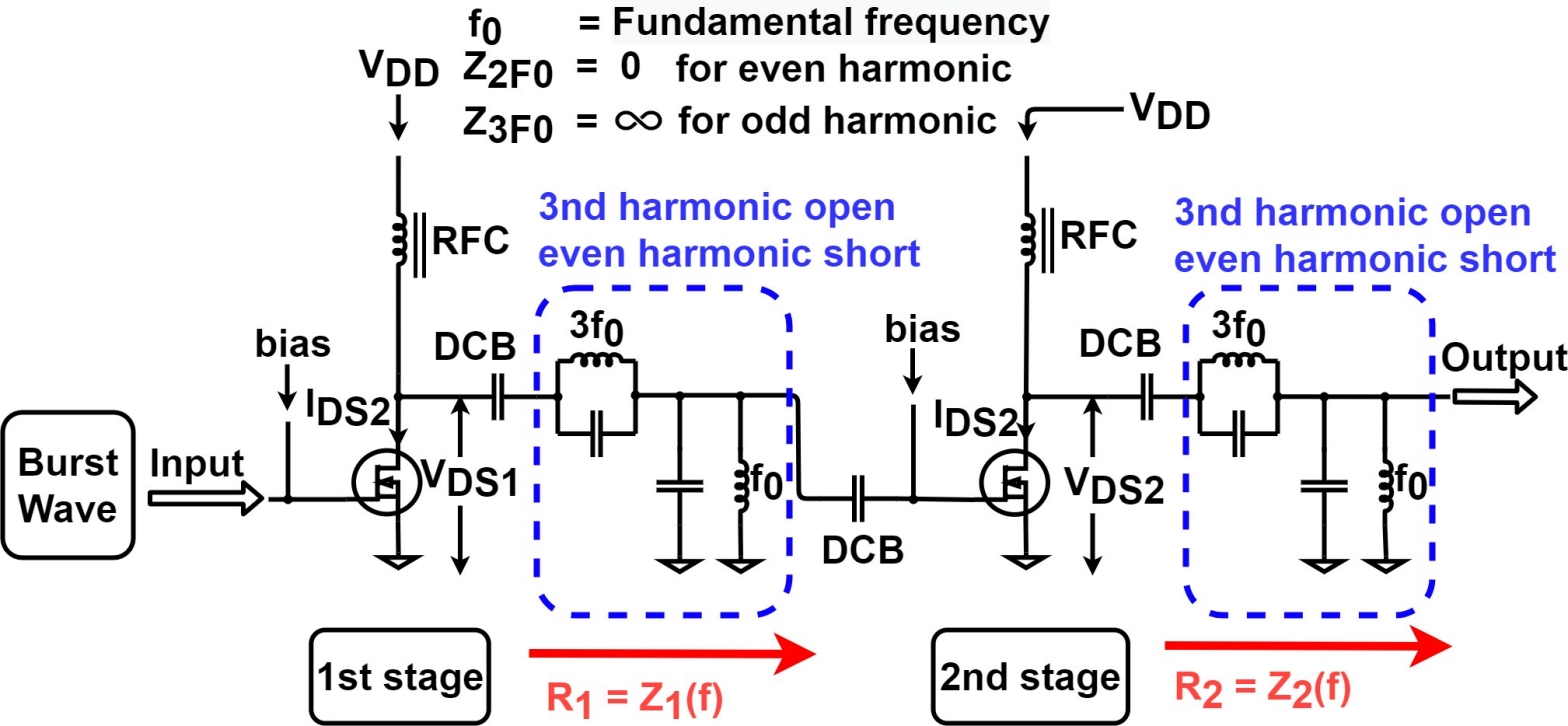
The ideal schematic diagram that is to calculate the drain efficiency of the two-stage class F amplifier which controlled only third harmonic.

Fig 4 is the simplified form of two-stage class F amplifier to calculate the maximum drain efficiency. As shown in Fig 4, we assume that each resonance circuit is completely open and shorts the signal, which corresponds to the resonance frequency. In Fig 4, we also assume that the impedances R1 and R2 of the first stage and the second stage are the same, and the overall drain efficiency can be expressed as follows:

$$\eta_{1stage}=\eta_{2stage}$$ (1)

$$\eta_{total}=\eta_{1stage}\times \eta_{2stage}={{(\eta }_{1stage})}^{2}$$ (2)

We assume that the class F amplifier has the form of an ideal current source with no loss and zero saturation voltage and output capacitance [32]. Then, we can approximate the drain efficiency of a class F power amplifier. The approximation of the drain efficiency, according to the control of the odd and even harmonics has been widely used in previous studies, and the data of approximation is obtained from the literature [32]. According to the class F theory, the drain efficiency of one stage can achieve maximum $\frac{9\pi }{32}$ = 88.4% when controlling only all even and third harmonics. Therefore, the combined drain efficiency of the two stages is ${\left(\frac{9\pi }{32}\right)}^{2}$, and a two-stage class F amplifier can achieve up to 78.1%.

**Fig 5:**
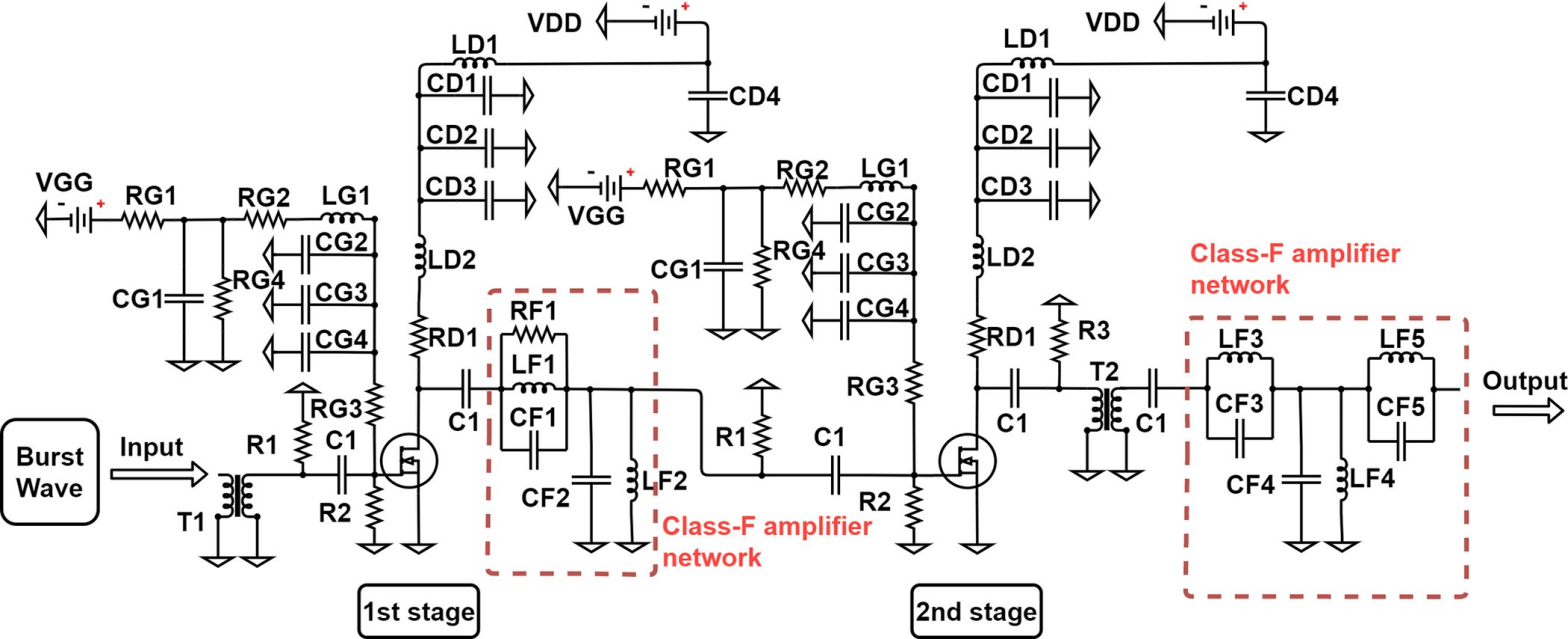
Designed two-stage high efficiency and high voltage class F amplifier schematic diagram.

Fig 5 shows the schematic diagram of the high-efficiency and high-voltage grade F amplifier we designed. Table 1 shows the element values of circuit elements in Fig 5. The amplifier was designed to consider various parameters, such as the output amplitude, distortion, and efficiency for wireless ultrasound systems [38]. The high-efficiency and high-voltage amplifier was designed to have two stages in order to reduce the load on the high-voltage transistor by considering each amplification factor to protect the transistor device and to guarantee a sufficient output power. In the first stage, only the third harmonic was controlled via CF1 and LF1. In the second stage, the third harmonic was controlled via CF3 and LF3, and the second harmonic was controlled via CF5 and LF5. However, the bandwidth of the resonance frequencies of CF1 and LF1 is very narrow, so RF1 was used to increase the bandwidth and this helps the voltage waveform get close to the square wave, the ideal class F voltage waveform. In addition, R3 is a damping resistor that helps to have a flat waveform by slowing the time the voltage is discharged. It is designed to reduce the remaining signals with the exception of the basic signal using parallel resonators composed of CF2 and LF2 with CF4 and LF4 in the first and second stages, respectively. The use of only CF4 and LF4 cannot completely eliminate the secondary harmonic in the second stage, and the output signal may contain the secondary harmonic. Therefore, the third harmonic was controlled using CF5 and LF5. In addition, low-frequency noise, which can be generated from the input signal or externally, can negatively affect the ultrasound images [39]. Large static electricity can damage other devices or may be fatal to ultrasound images. Therefore, using the transformers (T1, T2) having a 1:1 turns rate, the original signal passes through the input output stage and transmits static electricity and low-frequency noise to the ground. In addition, because R2 has a very large resistance value of 1 MΩ, it does not significantly affect the impedance, and helps to remove the residual noise remaining in the input signal.

**Table 1:** Numerical values of circuit elements in Fig 5.

Component	Value	Component	Value
R1	51 Ω	CD3	47 pF
R2	1 M Ω	CD4	220 μF
R3	100 Ω	CF1	68 pF
RG1	8.2 k Ω	CF2	100 pF
RG2	100 Ω	CF3	68 pF
RG3	18 kΩ	CF4	100 pF
RG4	2 kΩ (Potentiometer)	CF5	68 pF
RD1	100 Ω	LG1	1 μH
RF1	30 Ω	LD1	3.5 μH
C1	1800 pF	LD2	1 μH
CG1	220 μF	LF1	68 nH
CG2	0.01 μF	LF2	1000 nH
CG3	1000 pF	LF3	68 nH
CG4	47 pF	LF4	2000 nH
CD1	1000 pF	LF5	150 nH
CD2	0.01 μF		

#### Simulation based phase analysis

**Fig 6:**
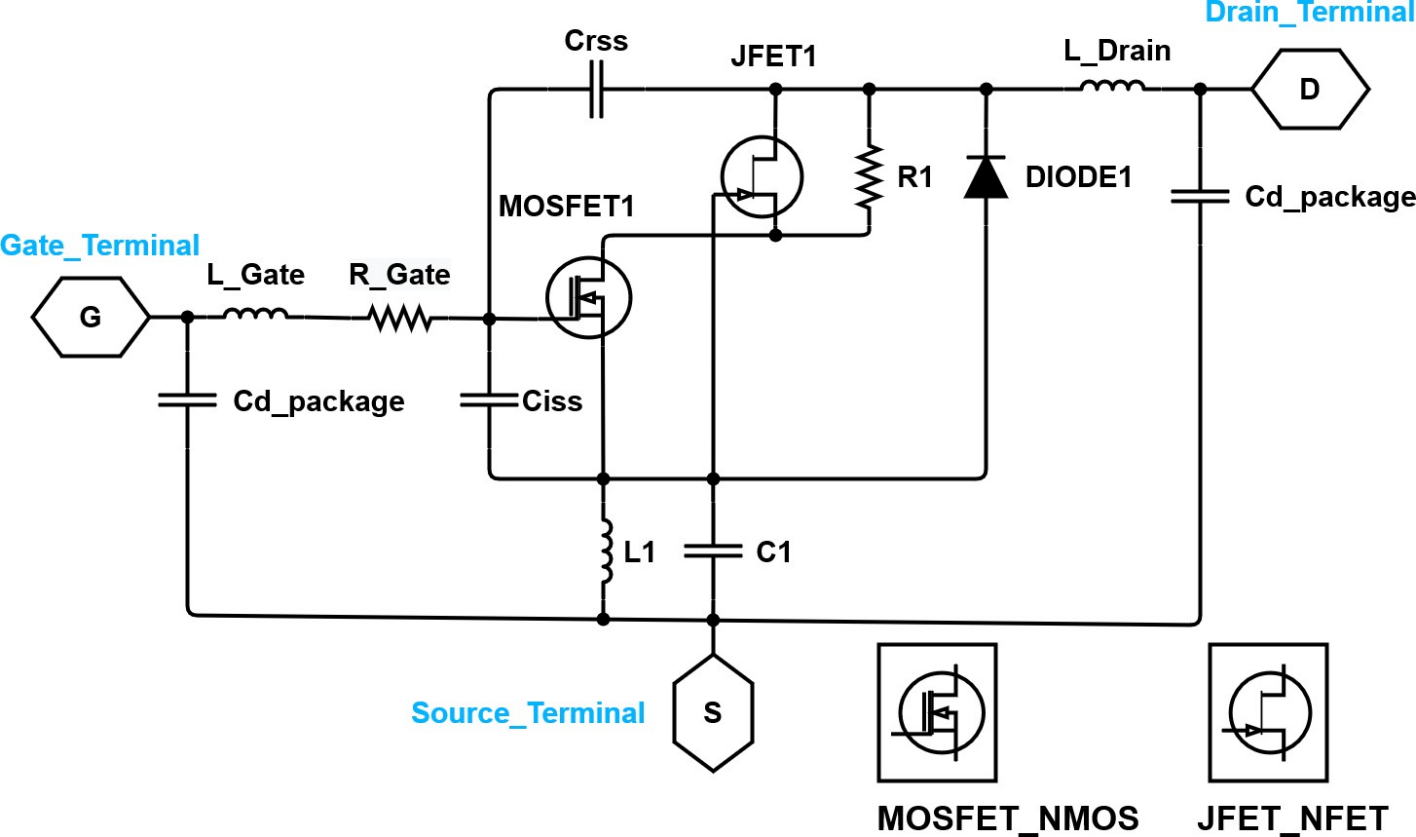
Large-signal LDMOSFET equivalent circuit provided by STMicroelectronics.

Analyses of the phases of the drain voltage and drain current were performed using the simulation software ADS. The circuit design of the simulation was tested based on Fig 5. In the software, the equivalent circuit of the high-voltage lateral double diffused metal-oxide semiconductor field effect transistor (LDMOSFET) provided by STMicroelectronics (see Fig 6) and the remaining elements of the scheme used the fundamental elements provided by the ADS software.

**Fig 7:**
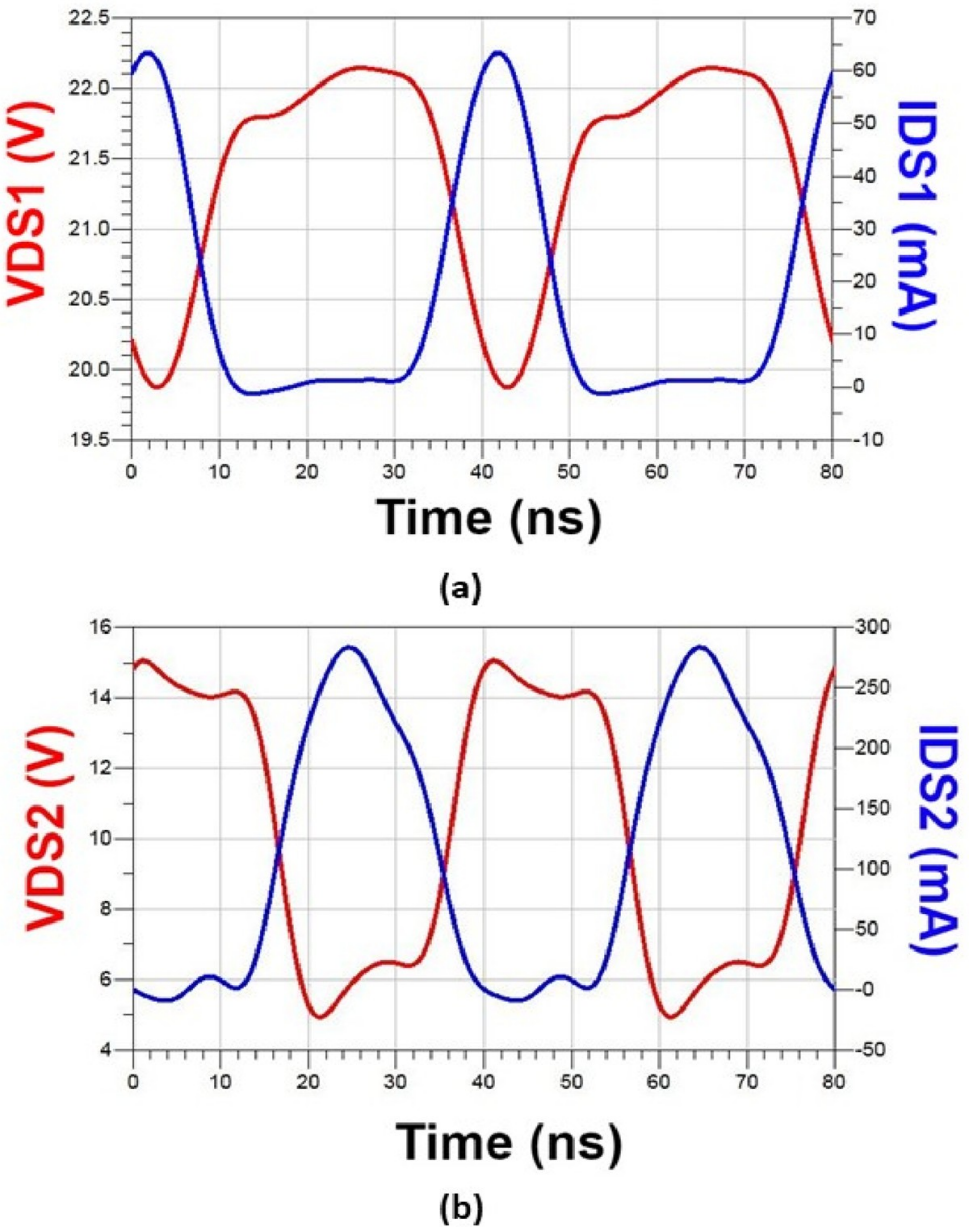
Simulation data of high-efficiency and high-voltage class F amplifier. Red and blue lines represent the drain voltage and drain current, respectively. The voltage and current waveform data from (a) the first stage and (b) the second stage.

**Fig 8:**
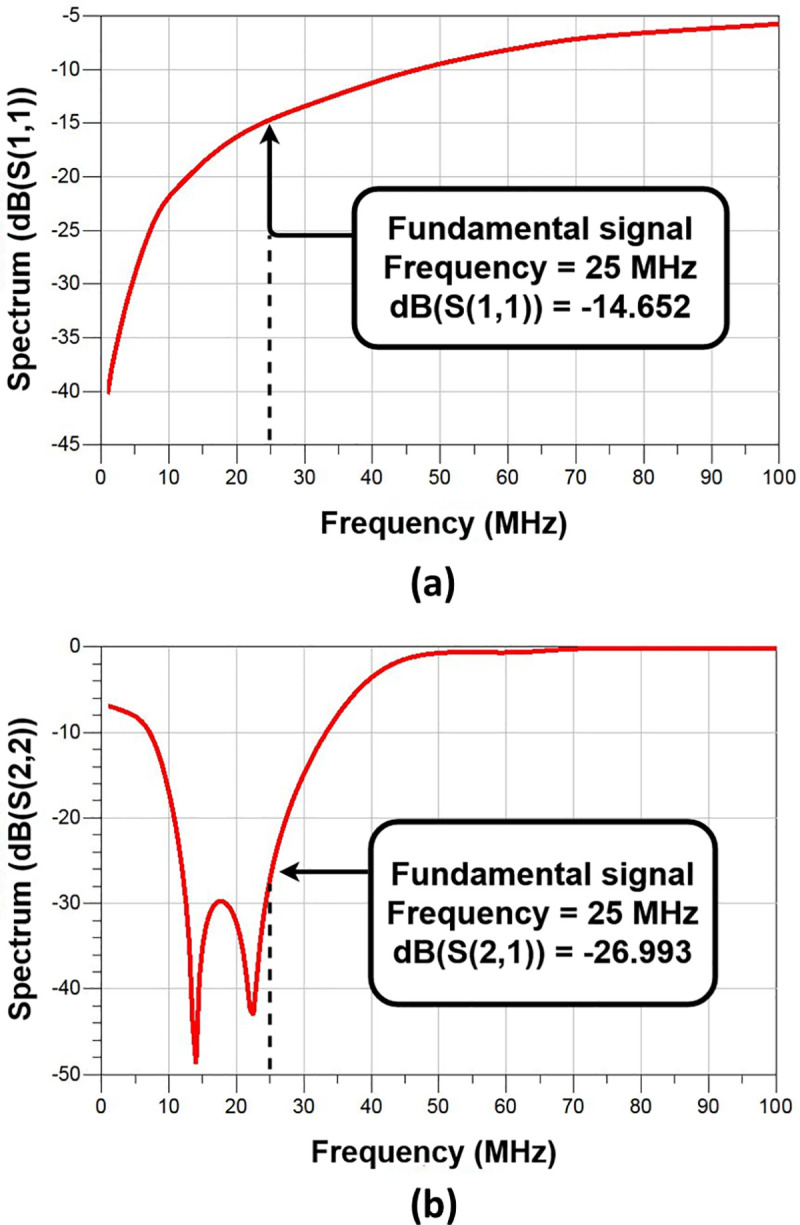
Simulation data of high-efficiency and high-voltage class F amplifier. S parameter used for impedance matching (a) S11, (b) S22.

The center frequency was 25 MHz and the harmonic components were varied using a resonant circuit composed of CF and LF (Class F amplifier network in Fig 5), and it was tuned to minimize the overlap of the drain source voltage and rectification waveform. The resonant frequencies of CF and LF were adjusted by simulating the S-parameters (see Fig 8), and the voltage and current of the drain source were analyzed to implement an appropriate high-efficiency and high-voltage class F amplifier. Fig 7 shows the drain source voltage and current waveforms in the simulation results. By varying the harmonic component, VDS2 and IDS2 have a slightly flat waveform. In addition, we can estimate that the high-efficiency and high-voltage class F amplifier operates properly by minimizing the overlapping period between the current and voltage waveforms.

The mismatch impedance between an amplifier and a transducer causes the loss in signal amplitude and waveform, thus reducing the signal to noise ratio and waveform distortions [40]. Therefore, electric impedance matching is needed to reduce signal loss and SNR due to mismatch impedance between the transducer and the amplifier. Furthermore, to maximize the power transfer between the ultrasound transducer and these devices, an electrical impedance matching is needed [40,41]. Accordingly, we did impedance matching. Fig 8 is the S-parameter simulation result through ADS software. The S(1,1) parameter was tuned to have the value less than −10 dB, thus it was −14.652 dB. In addition, the S(2,2) parameter was tuned to have a value less than −10 dB, thus it was −26.993 dB. In this way, the values of S(1,1) and S(2,2) were tuned to have appropriate values.

## Experiment environment and method

A high-efficiency and high-voltage class F amplifier was designed to operate at 25 MHz. Therefore, the variation of the output amplitude with frequency was analyzed to determine its suitability for use at 25 MHz. In addition, the class F amplifier and transducer were used together to analyze the THD and pulse echo signal waveforms [17]. We measured and analyzed the power, gain, power added efficiency (PAE), and DC current to estimate the performance based on the amplifier’s input signal in the appropriate frequency band. Owing to the capacitance, inductance, and resistance component values of the equivalent circuit model in the transducer with measurement equipment, the shift in the resonant frequency of the amplifier was considered [25].

### Amplifier performance measurement method

**Fig 9:**
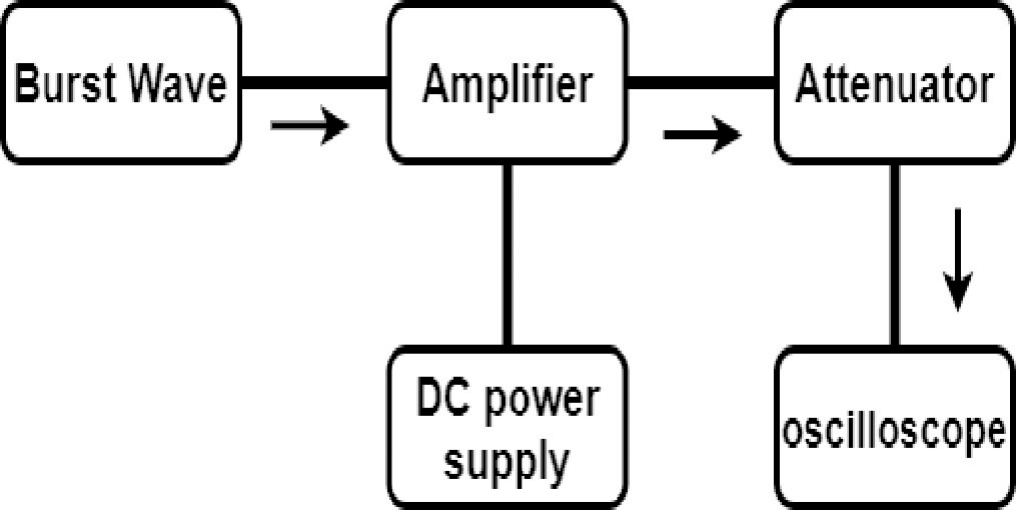
Block diagram showing amplifier performance measurement.

Fig 9 shows the measurement process used to determine the performance of the high-efficiency and high-voltage amplifier. The input signal was applied from the function generator to the power amplifier, and the DC power supply supplies the power to the gate and drain of the transistor. The signal amplified by the high-efficiency and high-voltage class F power amplifier was attenuated through the attenuator device, and was then shown on the oscilloscope.

**Fig 10:**
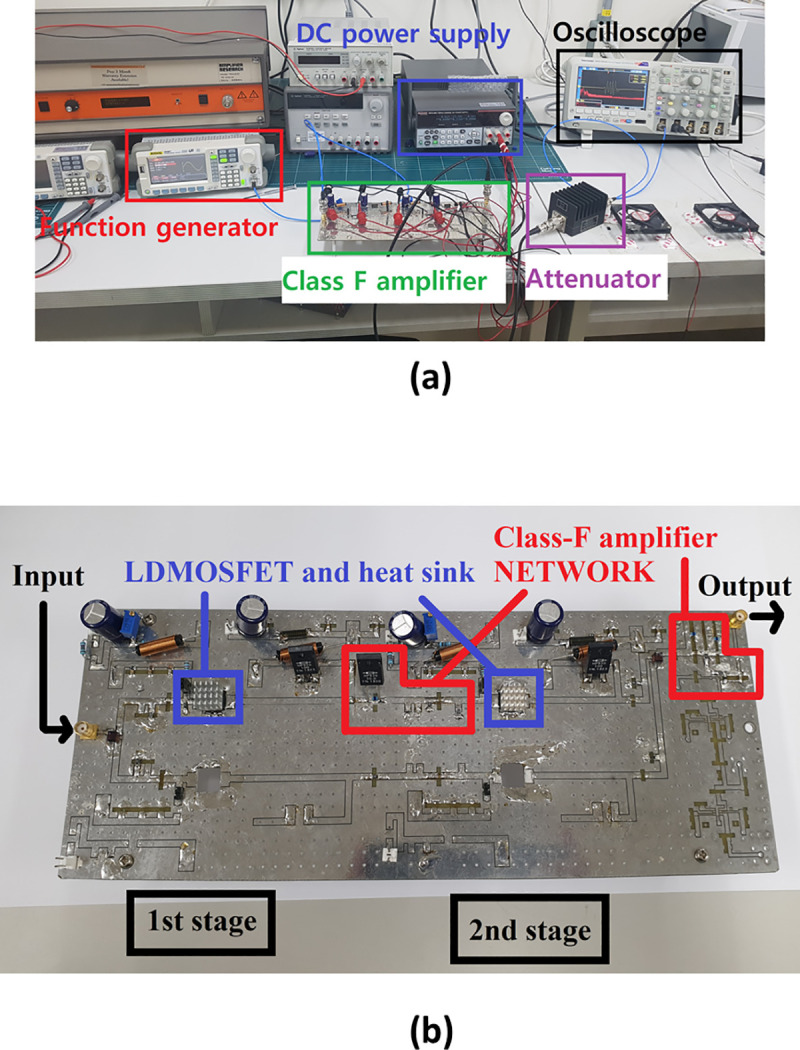
(a) Measurement environment for manufactured class F amplifier performance and (b) zoomed-in manufactured class F amplifier.

The experiment was performed according to the method shown in Fig 9. The DC power supply used in the experiment provides a voltage of 23.5 V each to the gate and drain sides of a two-stage high-efficiency and high-voltage class F amplifier. The input signal was provided through a function generator. A burst wave with a frequency range from 15 MHz to 35 MHz and three cycles was used as the input signal. The bias voltage was adjusted to 3 V using a variable resistor circuit. Amplified large signals can cause serious damage to the oscilloscope when an output voltage above 5V is applied. Therefore, an attenuator was used to protect the oscilloscope. The attenuator provides signal attenuation that is about 100 times less, which is 40 dB. Fig 10 shows the actual test environment with the measurement equipment.

In ultrasonic systems, acoustic signals are attenuated and scattered as they pass through the medium [42]. Therefore, an acceptable amplitude signal is required to obtain the signal data from the medium. The output amplitude checked in the oscilloscope was used to calculate the gain and PAE of the high-efficiency and high-voltage class F amplifier itself. The PAE is an important performance indicator that is used to describe the amount of input power used in the amplification process, and high-efficiency amplifiers can be the components in the system that save power and energy [20,29]. The amplifier PAE can be obtained by subtracting the input power from the output power and dividing the result by the DC power. Thus, the PAE indicates the amplifier efficiency because only the power used in the amplification process is expressed as the efficiency. The gain represents the amplification factor of the amplifier, and it is also one of the performance indicators of the amplifier [29]. In general, ultrasonic transducer probes have proprietary RF shielding for obtaining high signal-to-noise characteristics. However, if there are many harmonic components in the output signal of the amplifier, the harmonic components of the output signals are also transmitted to the echo signal and then, received through the transducer. In addition, the output signals of the PA with more than 10,000-cycle of pulse is utilized for the Doppler, coded excitation, neuromodulation, and acoustic stimulation applications. For these applications, harmonic performances are really in concern compared to general ultrasound imaging. These unwanted harmonic signals could affect the performances of the ultrasonic transducers, especially for high frequency ultrasound transducers. In addition, the velocity, reflection coefficient, and absorption coefficient of the medium are different because the harmonic components in the ultrasonic system have different fundamental signal and frequency characteristics. Therefore, the harmonic components must be minimized because they can provide distorted information. Harmonics above the fourth order have very low values. Therefore, the THD was calculated by measuring only up to the third harmonic. In the spectrum domain, we can determine a suitable frequency band by analyzing the THD values. In the time domain, the echo signals that are transmitted and received through the transducer help to show the suitability of the designed amplifier for ultrasonic systems.

### Pulse echo measurement method

**Fig 11:**
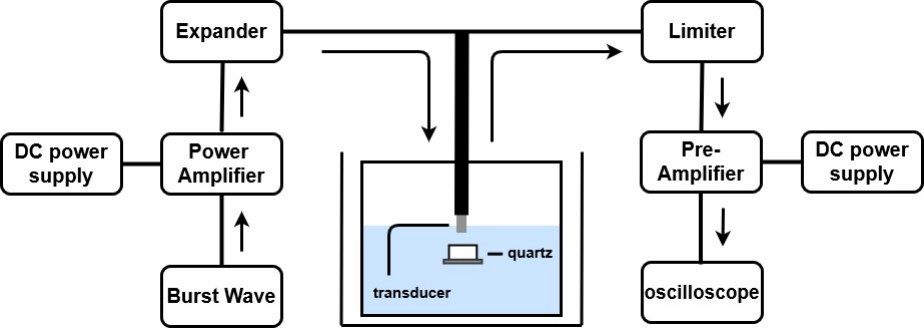
Ultrasonic pulse echo measurement process.

**Fig 12:**
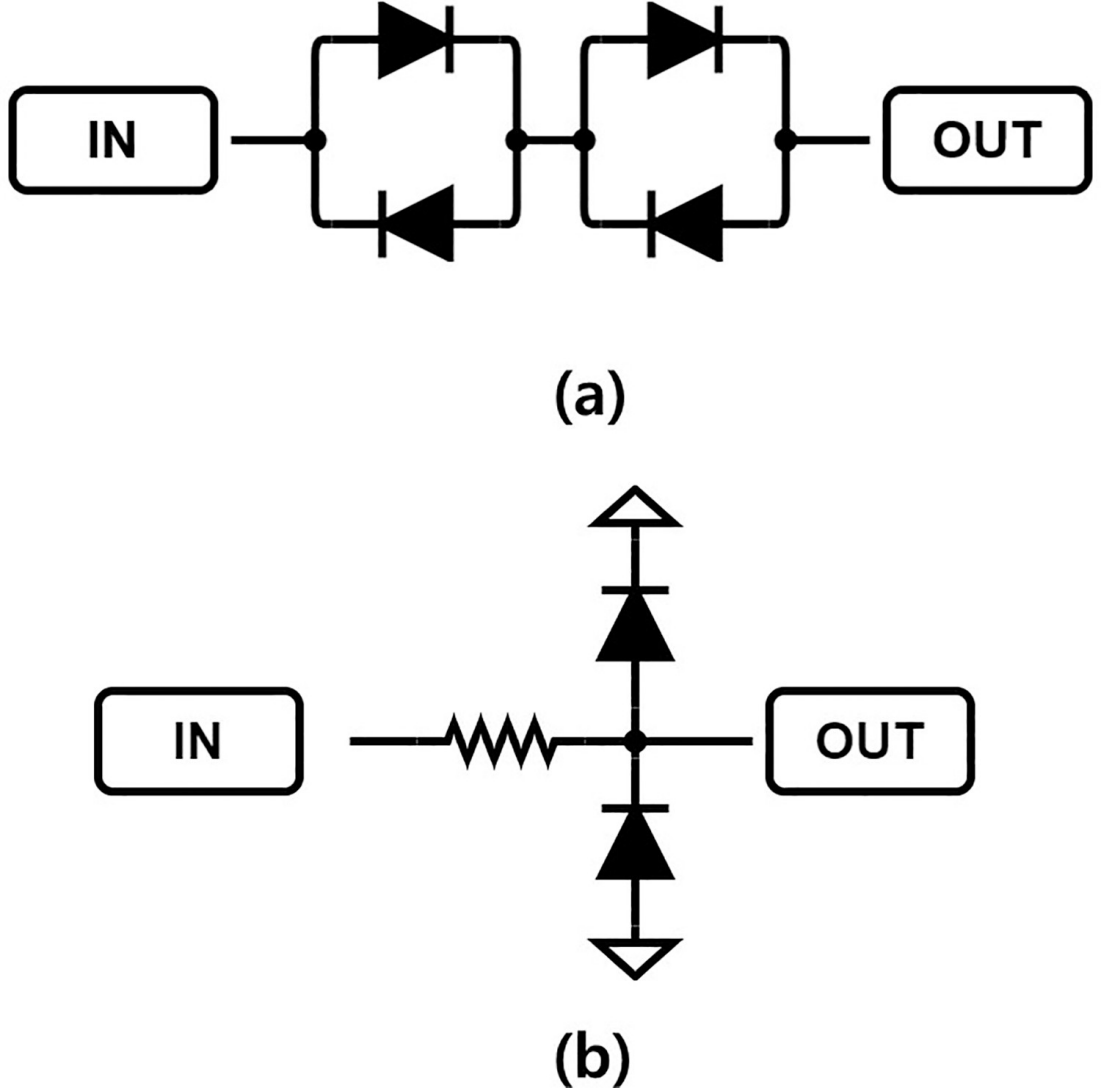
Limiter and expander schematic used in the experiment. (a) Expander (b) limiter circuits.

**Fig 13:**
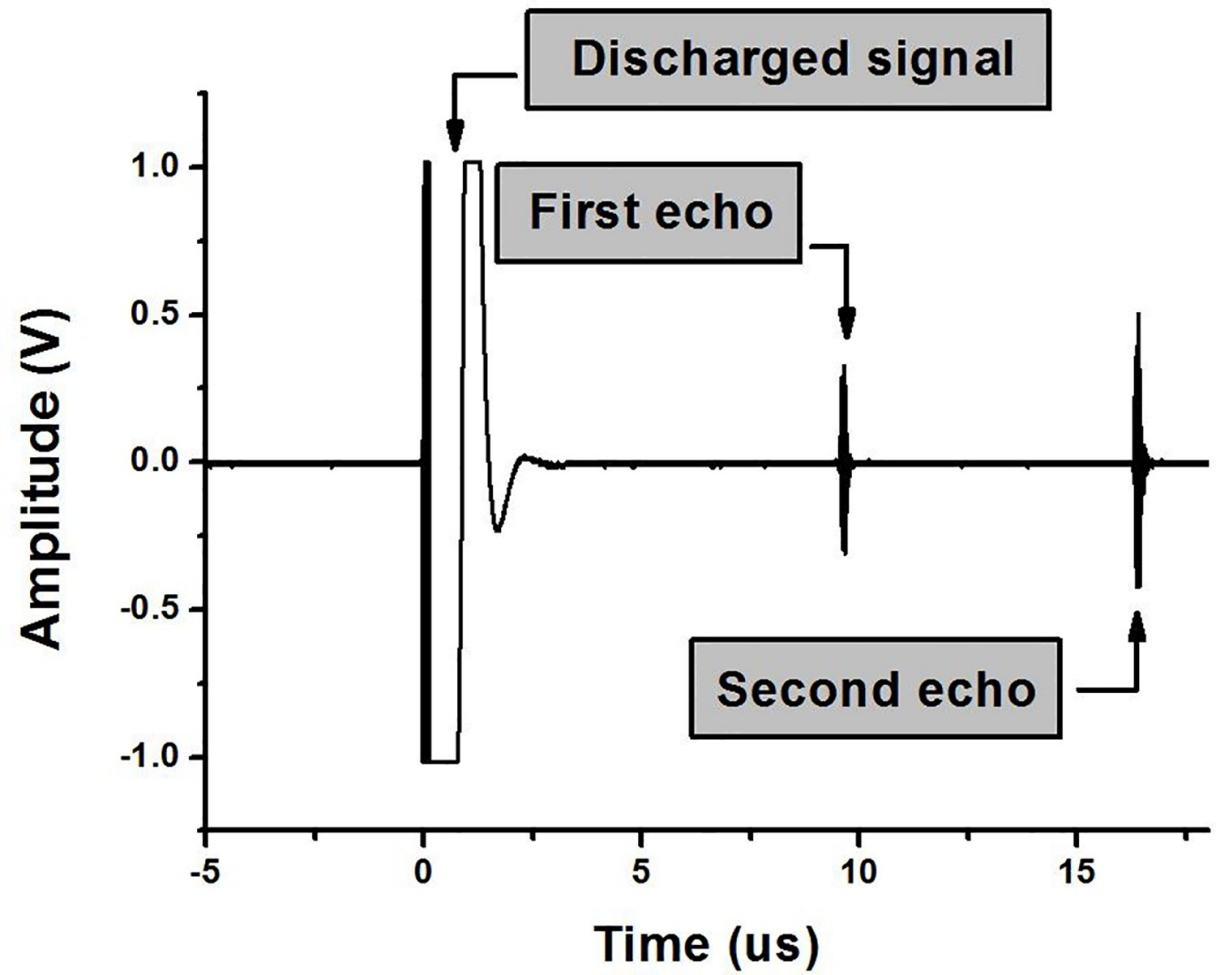
Discharged signal and ultrasonic echo signal transmitted and received by a high-efficiency and high-voltage class F amplifier and 25 MHz transducers.

The amplifier was designed and tested to determine the compatibility of the designed high-efficiency and high-voltage class F amplifier with the transducer. Fig 11 is a block diagram that shows the pulse echo measurement process. Non-linear devices generate more noise in high-frequency environments, and the efficiency of the ultrasound system during high-frequency operations decreases significantly at low bias supply voltages [21]. If nonlinear devices such as expanders and limiters are used, various factors need to be considered. Fig 12(A) shows the expander circuit, which consists of two pairs of diodes. The expander serves to remove noise at a voltage lower than 2V_TH_ and to minimize the undesirable ring-down signal in the ultrasound signal process [43,44]. The discharged signals and received echo signals coexist on the same line in the time domain, and are produced and received through the transducer (Fig 13). The echo signal is very small, so there is a need to amplify the signal through a preamplifier, and then analyze the signal using an oscilloscope. However, discharged signals with large amplitudes are also increased by the preamplifier, and may cause damage to the oscilloscope, so the amplitude magnitude of the discharge signal should be minimized through the limiter. In addition, the limiter can protect against strong static electricity that may occur inside or outside of the devices [45]. The equivalent circuit of the limiter is shown in Fig 12(B). Only a smaller signal level between −V_TH_ and +V_TH_ (threshold voltage of the diode used) signal passes through the limiter and the signal above the threshold voltage is shorted [15].

To measure the pulse echo signals through ultrasound transducer, the experiment was conducted based on the setup, as shown in Fig 11. The same function generator, oscilloscope, and DC power supply were used to measure the performance of the amplifier itself. The amplified signal was transmitted via the transducer after passing through the expander. The transmitted signal passes through the distilled water and is reflected from the quartz, after which it is received again through the same transducer [46]. The received signal passes through a limiter and is amplified through a pre-amplifier, and then appeared on the oscilloscope. The quartz reflects the transmitted signal by more than 99%, so it was used to accurately measure the transmitted signal characteristics of the amplifier and transducer [47]. The pre-amplifier has a gain of about 32 dB, and operates with 15-V DC voltages provided by a DC power supply.

Finally, the Q factor of the echo signal generated by the transducer is the ratio of the center frequency to the bandwidth. The Q factor can be obtained through the measured echo signal, and the value of is expressed by the following equation.

$$Q\;factor=\frac{Center\;frequency}{Bandwidth}$$ (3)

Q factor has no unit and it can be calculated with the Eq (3). There are various ultrasound applications such as imaging, acoustic stimulation, Doppler, and high intensity focused ultrasound applications. For different applications, different cycles of the pulses are used from the amplifier. The Q factor of the echo signals generated from the ultrasound transducers could be different according to the usage and it could be adjusted according to the number of pulse cycles. We designed the class F power amplifier for various possible ultrasound applications. The 3 cylces pulses generated from the class F power amplifier was the input for a 25 MHz transducer.

## Result

Fig 10(B) is a photograph of the manufactured two-stage high-efficiency and high-voltage class F amplifier. Because of the potential for damage to the device due to overcurrent, the power resistor (RD1 in Fig 5) in the drain side is used. Heat sinks were used to minimize the thermal errors that can occur in LDMOSFETs.

### Amplifier performance measurement and analysis according to frequency

**Fig 14:**
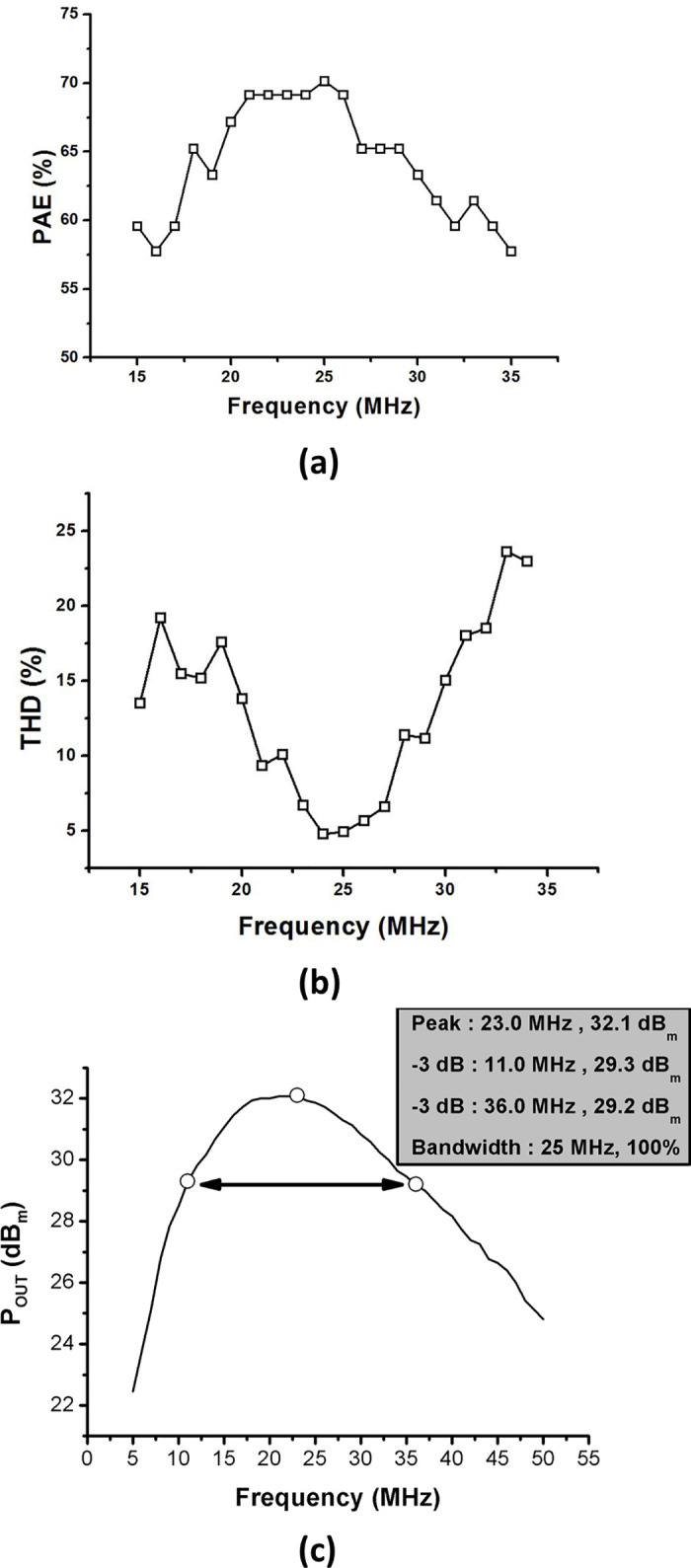
Curves showing variation of P_OUT_ and calculated PAE and THD values obtained from high-efficiency and high-voltage class F amplifier with a 7.5 dBm input power according to frequency. (a) PAE vs. Frequency, (b) THD vs. Frequency, (c) P_OUT_ vs. Frequency.

In order to measure the performance of the amplifier, the equipment shown in Fig 10 was used and measured in the same process as Fig 9. Fig 14(A) and 14(B) show the graphs of the PAE and THD with frequency. For frequency ranges from 15 MHz to 35 MHz, the THD and PAE are measured in increments of 1 MHz. In the experiment, the input was 7.5 dB_m_ when a burst wave signal of three cycles was applied. The input was applied equally for all frequencies from 15 MHz to 35 MHz, and a DC voltage of 23.5 V was applied to the gate and drain sides of the LDMOSFETs in the two-stage high-efficiency and high-voltage class F amplifier, respectively. The DC current was measured as 121 mA regardless of the frequency when the bias point and drain were applied to the LDMOSFET and the input three cycles burst wave was applied. At 25 MHz, the THD was achieved 5.0% and the PAE was the highest at 69.6%. The PAE was calculated based on the output signal. Therefore, at 25 MHz, the harmonic component may be included in the PAE calculation. Fig 14(C) shows a graph of P_OUT_ according to frequency variances. The -3 dB bandwidth points are 11.0 MHz and 36.0 MHz so the developed PA has 100% bandwidth.

### Ultrasonic pulse echo measurement and analysis

**Fig 15:**
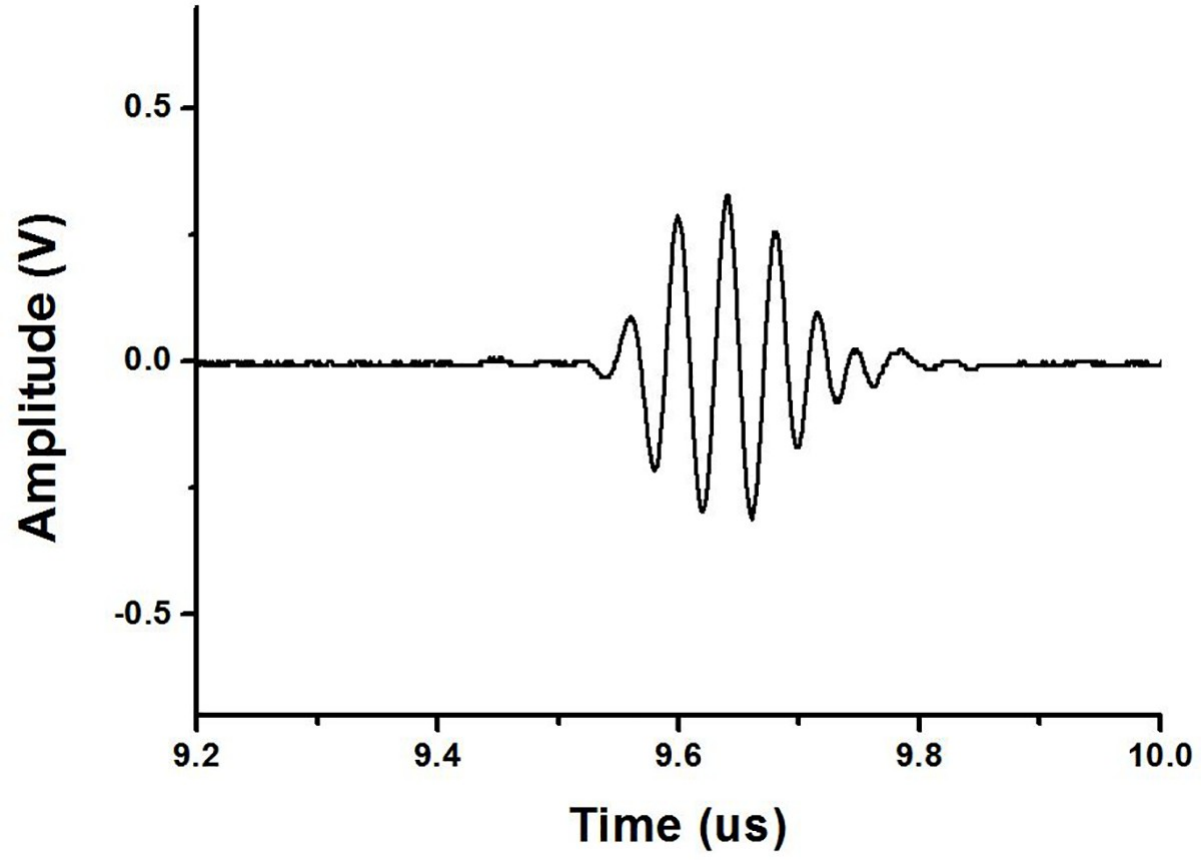
The first echo signal transmitted and received by a high-efficiency and high-voltage class F amplifier and 25-MHz transducers.

**Fig 16:**
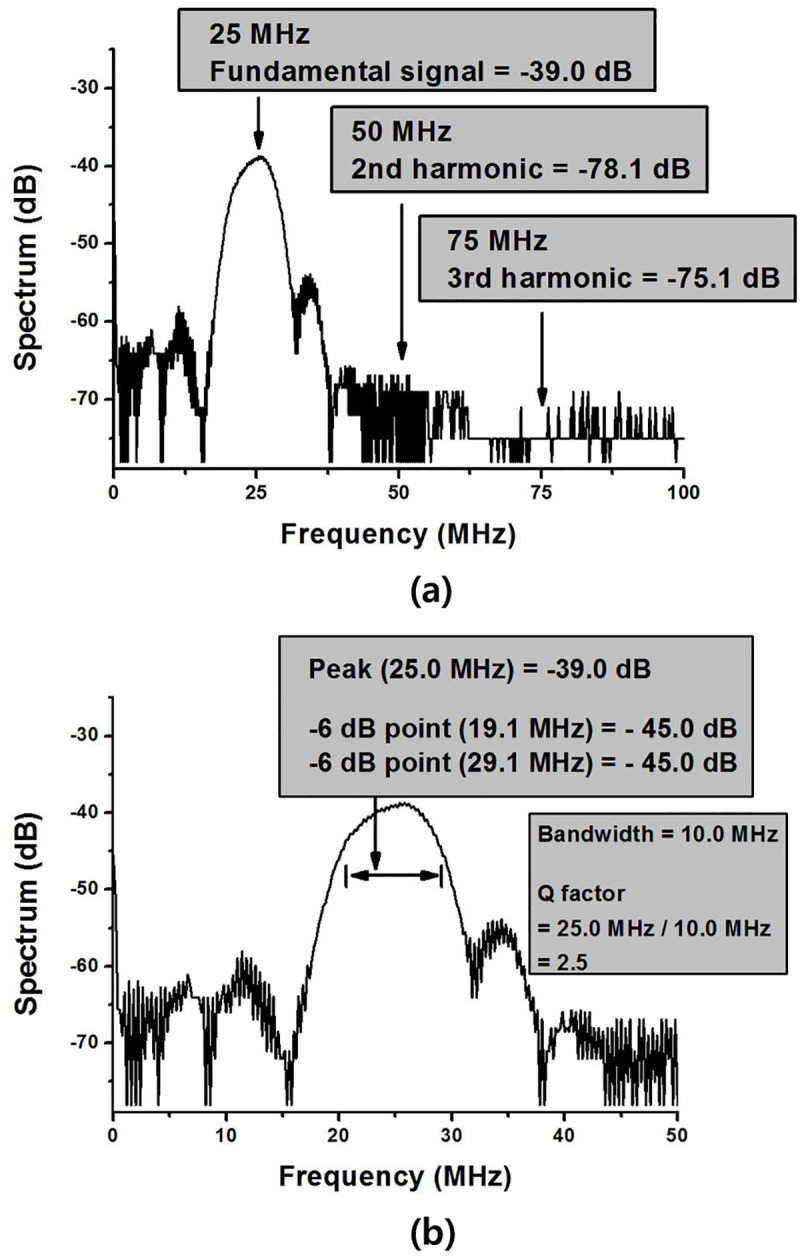
FFT spectrum data transmitted and received by a high-efficiency and high-voltage class F amplifier and 25-MHz transducers. (a) FFT spectrum data for fundamental signal, second harmonic and third harmonic (b) FFT spectrum data enlarged to show bandwidth.

Pulse echo signals were measured and analyzed using the 25-MHz ultrasonic transducer. Fig 15 shows the output signal waveform on the oscilloscope using a 25 MHz, three cycles burst wave as an input and a 25-MHz transducer. In addition, the FFT was applied to the waveform of the echo signal to show the spectrum data, as shown in Fig 16. The piezoelectric element in the ultrasound transducer cannot stop immediately if it vibrates using electric power. The increased number of cycle with longer durations due to the ring down negatively affects the axial resolution of the ultrasound image [13]. The characteristics of the piezoelectric element and the increased cycle with long durations owing to the harmonic component, negatively affect the axial resolution of the ultrasound image [25,42]. The axial resolution is a numerical indicator of the maximum image size, which is estimated by the reflected signal in the direction in which sound propagates [1]. The spatial pulse length is determined by the wavelength and the number of cycle of the pulse, and it is proportional to the axial resolution. The smaller the axial resolution, the clearer the image size, so it is necessary to minimize the pulse duration. At 25 MHz, a slight ring-down phenomenon was observed. Owing to the characteristics of the piezoelectric element, there was a very small ring down resulting from other causes except the increased number of cycle, so we obtained a relatively good waveform.

Because the attenuation and reflection coefficients in the medium are frequency dependent, and the absorption and scattering coefficients are also different in the medium, it is complicated to analyze the information from the medium even if higher harmonic components are present in the received echo signals. Therefore, in general ultrasound systems, it is necessary to minimize the harmonic components to obtain clear information. Fig 16(A) shows the FFT spectrum of the 25 MHz echo signals. The harmonic components were analyzed in the FFT spectrum. In the FFT spectrum, the fundamental signal (−39.0 dB), second harmonic (−78.1 dB), and third harmonic (−75.1 dB) were measured. The THD was calculated to be 4.5%. Table 2 shows the spectrum data at 25 MHz, as shown in Fig 16(A). The difference between the fundamental signal and the second harmonic is 39.1 dB, and the difference between the fundamental signal and the third harmonic is 36.1 dB. Fig 16(B) is the same data as Fig 16(A) and is an enlarged picture to see the bandwidth. The highest value was measured at –39.0 dB at 25.0 MHz. The frequency of -45.0 dB, which is the point of –6 dB, was measured at 19.1 MHz and 29.1 MHz. Therefore, the amount of bandwidth is 10.0 MHz and the Q factor is 2.5.

**Table 2:** Ultrasonic echo signal harmonics.

Frequency (MHz)	Fundamental signal (dB)	2^nd^ harmonic (dB)	3^rd^ Harmonic (dB)	THD (%)
25	–39.0	–78.1	–75.1	4.5

### Amplifier performance measurement and analysis at 25 MHz

**Fig 17:**
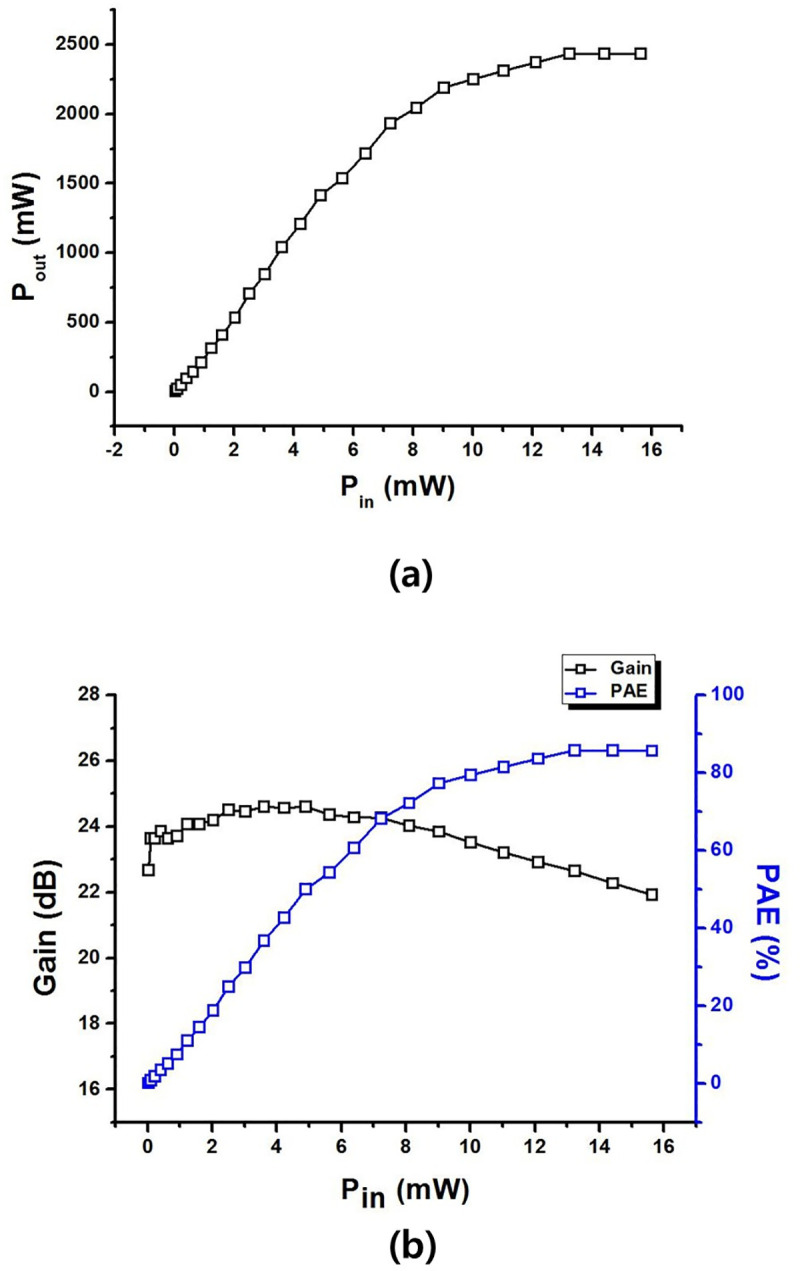
(a) Measured Pout and (b) calculated PAE and gain graphs from high-efficiency and high-voltage class F amplifier with an input frequency of 25 MHz.

Fig 17(A) and 17(B) shows P_OUT_ according to the input signals and shows the GAIN and PAE according to the input signals. In order to measure the ultrasonic echo signal, measured in the same process as Fig 11. To measure the amplifier performance at 25 MHz, we used the setup illustrated in Fig 9. The experimental environment is identical to the amplifier performance measurement and analysis previously shown in Fig 10. The DC current was measured as 121 mA when the bias point and drain were applied to the LDMOSFET and the input three cycles burst wave was applied when the input signal was varied from −16 dB_m_ to 10 dB_m_. The maximum output power was about 33.9 dBm and the PAE was 85.1%. The maximum voltage gain was 24.6 dB, at which time the output power (Pout) was 1040 mW. In addition, the output P1dB was 2250 mW, and the PAE at that output P1dB was about 78.8%.

### Recent studies of ultrasonic amplifiers

Table 3 shows the summarized performances of class AB amplifier, class C amplifier, class F amplifier for ultrasound and other applications with our developed class F amplifier. These amplifiers may have used for different equipment applications. The amplifiers for ultrasound images are mainly used for wired-type ultrasound products so they utilize highly linear class A or class AB topology. It is difficult to apply class A or class AB topology to wireless ultrasound systems. What we mean is that it is difficult to design a wireless ultrasound system due to the low efficiency of the amplifier and the limited battery. This paper first applied the amplifier using efficient class F topology to ultrasonic diagnostic application such that the results were analyzed. Since there are a few non-linear amplifier research works for ultrasound systems, the contents contained in Table 3 do not show the amplifier design for wireless ultrasound systems.

**Table 3:** Summarized performances of class AB amplifier [48], class C amplifier [49], class F amplifier [50,51], and our developed class F amplifier.

Parameters	Class AB	Class C	Class F	Class F	Class F (Our work)
Output	60 [V_p-p_]	32.9 [dB_m_]	40.0 [dB_m_]	18 [dB_m_]	33.5 [dB_m_]
Gain			16.9 [dB]	18 [dB]	23.5 [dB]
Frequency	15 [MHz]	25 [MHz]		2.4 [GHz]	25 [MHz]
PAE	-	-	68.0 [%]	46 [%]	78.8 [%]
Efficiency	-	-	69.4 [%]		-
THD	< 3.5 [%]	-			5.0 [%]
Echo bandwidth	-	18.25 [%] (@ 5 cycles)			40 [%] (@ 3 cycles)
Echo THD	7.76 [%] (@ 10 MHz)				4.5 [%]
Application	Piezoelectric transducer	Piezoelectric transducer		Wireless sensor network	Piezoelectric transducer

In class AB amplifier, the THD is less than 3.5% at 15 MHz and the Echo THD is 7.76% at 10 MHz. Ultrasonic echo THD was tested using commercial transducer probes with a central frequency of 10 MHz. In the Class C amplifier, the echo bandwidth was measured at 18.25% using 5 cycles of the input signal. In the class F amplifier we worked on, ultrasonic echo bandwidth was measured at 18.25% using 3 cycles of the input signal.

## Conclusion

In ultrasound systems, the high-voltage amplifier in the transmitter has very high power consumption. In particular, for wireless ultrasound systems, it is essential to achieve high efficiency by minimizing power consumption from the high-voltage amplifier. In addition, medical ultrasound systems must provide high-quality images to accurately diagnose the patient’s condition. To provide a high-quality video image, low distortion must be achieved using harmonic control. To achieve high efficiency and low distortion, a class F amplifier type was selected for wireless ultrasound systems. The designed high-voltage class F amplifiers can achieve high efficiency owing to the switching LDMOSFET and low distortion using the resonant circuit placed on the output side of the amplifier. In addition, each resonator can precisely control a specific harmonic component, thereby tuning the output voltages that are suitable for wireless ultrasound systems.

We designed a high-efficiency and high-voltage class F amplifier, analyzed the appropriate frequency bands, and then measured its performance. The designed high-efficiency and high-voltage class F amplifier can be used in frequency bands of 15 MHz to 35 MHz. Some trade-offs are required among the various characteristic elements in the amplifier, such as the gain, PAE and THD, and resonant frequency, which need to be properly tuned to achieve performance suitable for wireless ultrasound systems. The higher the PAE, the higher the efficiency, but THD must be considered because harmonic components may be included. However, at 25MHz, the PAE is high enough at 69.6%, and the THD is also low at 5.0%. In addition, except for the phenomenon due to the characteristics of the piezoelectric element, it did not have a significant effect on the pulse width of the waveform of the echo signal, and the echo signal THD is a sufficiently low value of 4.5%. In addition, at 25 MHz, the echo signal has a wide bandwidth of 10.0 MHz, and the Q factor is calculated as 2.5. Consequently, the designed high-efficiency and high-voltage class F amplifier shows good performance that can be used for ultrasonic diagnostics.

DC current is a direct and critical factor that increased electronic and system temperature. Performance can be varied as the temperature of the element increases. Therefore, an external cooling fan is essential to lower the temperature using a large size heat sink. As a result, the DC current should be lowered to reduce the burden of wireless ultrasound systems and to minimize temperature-dependent errors. The LDMOSFET used in this amplifier is a high-voltage active element, whose performance depends on the temperature change. However, the measured DC current used in this class F amplifier for different amplitudes or frequency ranges is the same with a value of 121 mA. Nevertheless, to minimize errors related to variations in temperature, the experiment was conducted with a heat sink which was applied to the LDMOSFETs. In fact, the use of a high-efficiency and low-DC current amplifier can reduce costs by minimizing external cooling fan system and portable battery volumes. Therefore, a more compact design will help diagnostics longer and more conveniently.

## Supporting information

S1 Table Amplifier performance measurement results. (DOCX)

S2 Table Amplifier performance at 25 MHz. (DOCX)
