# Supplementary material for: High-efficiency high-voltage class F amplifier for high-frequency wireless ultrasound systems
Source: PLoS One. 2021 Mar 29;16(3):e0249034. doi: 10.1371/journal.pone.0249034 (PMC8006987; doi:10.1371/journal.pone.0249034)
Supplement: S1 Table — (DOCX) [file pone.0249034.s001.docx]

**S1 Table. Amplifier performance measurement results.**

| **Frequency**  **(MHz)** | **Current**  **(ma)** | **PAE**  **(%)** | **THD**  **(%)** |
| --- | --- | --- | --- |
| 15 | 121 | 59.1 | 13.5 |
| 16 | 121 | 57.3 | 19.2 |
| 17 | 121 | 59.1 | 15.5 |
| 18 | 121 | 64.7 | 15.2 |
| 19 | 121 | 62.8 | 17.6 |
| 20 | 121 | 66.6 | 13.8 |
| 21 | 121 | 68.6 | 9.4 |
| 22 | 121 | 68.6 | 1.01 |
| 23 | 121 | 68.6 | 6.7 |
| 24 | 121 | 68.6 | 4.8 |
| 25 | 121 | 69.6 | 5.0 |
| 26 | 121 | 68.6 | 5.7 |
| 27 | 121 | 64.7 | 6.6 |
| 28 | 121 | 64.7 | 11.4 |
| 29 | 121 | 64.7 | 11.2 |
| 30 | 121 | 62.8 | 15.1 |
| 31 | 121 | 60.9 | 18.0 |
| 32 | 121 | 59.1 | 18.5 |
| 33 | 121 | 60.9 | 23.7 |
| 34 | 121 | 59.1 | 23.0 |
| 35 | 121 | 57.3 | 24.5 |
