# Supplementary material for: High-efficiency high-voltage class F amplifier for high-frequency wireless ultrasound systems
Source: PLoS One. 2021 Mar 29;16(3):e0249034. doi: 10.1371/journal.pone.0249034 (PMC8006987; doi:10.1371/journal.pone.0249034)
Supplement: S2 Table — (DOCX) [file pone.0249034.s002.docx]

**S2 Table. Amplifier performance at 25 MHz.**

| **Pin**  **(dB_m_)** | **Current**  **(mA)** | **Pout**  **(dB_m_)** | **Gain**  **(dB)** | **PAE**  **(%)** |
| --- | --- | --- | --- | --- |
| –16.0 | 121 | 6.7 | 22.7 | 0.2 |
| –10.0 | 121 | 13.6 | 23.6 | 0.8 |
| –6.5 | 121 | 17.2 | 23.6 | 1.8 |
| –4.0 | 121 | 19.9 | 23.9 | 3.4 |
| –2.0 | 121 | 21.6 | 23.6 | 5.1 |
| –0.5 | 121 | 23.3 | 23.7 | 7.4 |
| 0.9 | 121 | 25.0 | 24.1 | 11.0 |
| 2.0 | 121 | 26.1 | 24.1 | 14.3 |
| 3.1 | 121 | 27.3 | 24.2 | 18.7 |
| 4.0 | 121 | 28.5 | 24.5 | 24.7 |
| 4.8 | 121 | 29.3 | 24.5 | 29.7 |
| 5.6 | 121 | 30.2 | 24.6 | 36.5 |
| 6.3 | 121 | 30.8 | 24.6 | 42.4 |
| 6.9 | 121 | 31.5 | 24.6 | 49.6 |
| 7.5 | 121 | 31.9 | 24.4 | 53.9 |
| 8.1 | 121 | 32.3 | 24.3 | 60.1 |
| 8.6 | 121 | 32.9 | 24.3 | 67.7 |
| 9.1 | 121 | 33.1 | 24.0 | 71.6 |
| 9.6 | 121 | 33.4 | 23.9 | 76.7 |
| 10 | 121 | 33.5 | 23.5 | 78.8 |
| 10.4 | 121 | 33.6 | 23.2 | 80.9 |
| 10.8 | 121 | 33.8 | 22.9 | 83.0 |
| 11.2 | 121 | 33.9 | 22.6 | 85.1 |
| 11.6 | 121 | 33.9 | 22.3 | 85.1 |
| 11.9 | 121 | 33.9 | 21.9 | 85.0 |
